# Psychosocial distress and functioning of Greek youth with cystic fibrosis: a cross-sectional study

**DOI:** 10.1186/1751-0759-8-13

**Published:** 2014-06-11

**Authors:** Konstantina Kostakou, George Giannakopoulos, Stavroula Diareme, Chara Tzavara, Stavros Doudounakis, Stelios Christogiorgos, Chryssa Bakoula, Gerasimos Kolaitis

**Affiliations:** 1 Department of Child Psychiatry, Athens University Medical School, Aghia Sophia Children’s Hospital, Athens, Greece; 2 Cystic Fibrosis Unit, Aghia Sophia Children’s Hospital, Athens, Greece; 3 Athens University Medical School, Athens, Greece

**Keywords:** Children and adolescents, Cystic fibrosis, Family functioning, Psychopathology, Self-esteem, Social adjustment

## Abstract

**Background:**

To assess psychosocial functioning and distress of children and adolescents with cystic fibrosis compared to healthy controls.

**Methods:**

Thirty-six patients with cystic fibrosis aged 8–18 years (24 boys, mean age ± SD: 11.5 ± 2.6 years) and 31 sex- and age-matched healthy control subjects (18 boys, mean age ± SD: 12 ± 2.5 years) were enrolled in the study. In order to assess the self-esteem, social adjustment, and family functioning of these young people, the Culture-free Self-esteem Inventory, the Social Adjustment Scale–Self-Report, and the Family Assessment Device were administered. Emotional/ behavioral problems were assessed through the Youth Self Report and the Child Behavior Checklist given to both the subjects and their parents.

**Results:**

No significant differences were found for self-esteem between the two study groups. Regarding social adjustment, children with cystic fibrosis reported significantly worse friendship and overall adjustment (*P* < 0.05). Moreover, no difference was found in the levels of family functioning between the two groups. No significant differences between the groups were found in emotional/ behavioral problems from the self-reports. On the contrary, parents of children with cystic fibrosis reported significantly higher levels of withdrawal/ depression, thought problems, and delinquent behavior (*P* ≤ 0.01) as compared to controls.

**Conclusions:**

Children and adolescents with cystic fibrosis appear to be a psychosocially vulnerable group. A biopsychosocial approach should emphasize the assessment and treatment of the psychosocial distress of these patients alongside multiple somatic treatments.

## Background

There is evidence that individuals with chronic medical conditions are at increased risk of psychiatric disorders and psychological distress [1-4]. Towards this end, there is a growing interest in psychosocial functioning among populations suffering from chronic diseases. Several studies have demonstrated that the risk for psychiatric problems in children with chronic medical conditions is about two to four times greater than that in their healthy peers [2,5]. Symptoms of anxiety and depression as well as behavioral problems appear to be more common in populations with chronic conditions than in the general population [6-8]. Additionally, the improvement in survival rates of most of the chronic conditions results in a growing need for further detection and evaluation of the psychological well-being of patients.

Cystic fibrosis (CF) is an example of a chronic illness with an improving survival rate. It is the most common life-limiting genetic disorder in white people, with a frequency of about 1 in 3500 live births [9,10], affecting almost 50.000 individuals in the European Union, with a mean prevalence of 0.737/10.000 [11]. Life expectancy of CF patients is rising towards a mean of 40 years with advances in all aspects of therapy apart from treating the basic molecular defect [12,13]. CF is characterized by chronic airway obstruction and subsequent infection of the bronchial airways. Respiratory symptoms are experienced by nearly every patient. Gastrointestinal consequences include exocrine pancreatic insufficiency (in an estimated 90% of individuals), which sets the stage for CF-related diabetes in an estimated 50% of adults over the age of 30 years. Infertility is present for nearly 95% of males due to absence of the vas deferens and for 20% of females due to ion transport issues in the genitourinary system, such as abnormal cervical mucus [14]. Given the chronic, progressive and disabling nature of CF, multiple treatments are required on a daily basis.

Developing a sense of self and acquiring autonomy in all areas of life are the major developmental tasks throughout childhood. However, normal developmental issues of puberty, autonomy, identity acquisition, sexuality and education may become more difficult tasks to deal with in CF patients [15,16]. Children’s identity and sense of competence begin to be developed through a process of comparison with their peers [17]. Children with CF though have reduced growth velocity and delayed adolescent growth spurt [18], and such a comparison process may highlight their CF-related differences. Treatment burden, shortened mortality, and amplification of normal developmental issues may have an impact on the psychosocial well-being of young patients.

Consequently, there are numerous studies focusing on the psychosocial aspects of CF, but the results have been variable. There is some evidence indicating that the psychological and psychosocial functioning of CF patients are similar to that of the healthy population [19-21]. However there is also evidence that pediatric patients do suffer an increased likelihood of psychiatric problems and psychological distress [22-24]. More specifically, although anxiety and depression appear to be more common in patients with CF than in the general population, symptoms of anxiety occur more frequently than depression in this population [25].

As far as the emotional needs of the young patients with CF are concerned, selection of informant appears to be an important variable that should be taken into consideration when those needs are being evaluated. More specifically, in parental ratings depressive symptoms [26] and behavior problems appear to be more frequent than in self ratings [6].

In Greece, there are almost 800 patients and the frequency of the disease is 1 in 3.500 live births, similar to that in the European Union [11]. The aim of the present study was the evaluation of the psychosocial functioning of Greek children and adolescents with CF. Family functioning, self-esteem, social adjustment, and emotional/behavioral problems were examined and rates were compared to those of a group of healthy children and adolescents. The psychosocial functioning of children and adolescents with CF was expected to be worse than that of their healthy peers. Additionally, it was hypothesized that progression of age in the CF population might be followed by deterioration in emotional and behavioral functioning. Finally, parents and children were expected to show differences in their perceptions about the emotional/behavioral difficulties of these young patients.

## Methods

### Participants and procedures

Thirty-six patients with CF aged 8–18 years (24 boys, mean age ± SD: 11.5 ± 2.6 years) and 31 sex- and age-matched healthy control subjects (18 boys, mean age ± SD: 12 ± 2.5 years) were enrolled in the study. All patients were followed-up as outpatients at regular intervals after the diagnosis at the CF Unit of Aghia Sophia Children’s Hospital, Athens, Greece. The control subjects were recruited from a community sample and were healthy children without a sibling suffering from a chronic disease. The study was approved by the Scientific and Ethics Committees of Aghia Sophia Children’s Hospital, and all parents provided written informed consent before entry of their children in the study.

### Measures

All patients presented with good respiratory functioning (Forced Expiratory Volume- FEV% 91.15 for girls/89.2 for boys), as assessed by spirometry on the day of the data collection. More specifically, 31 of our patients had mild pulmonary impairment (FEV% predicted 70–100) and 5 of them had moderate pulmonary impairment (FEV% predicted 40–70). None of our participants was assessed with a severe pulmonary impairment (FEV% predicted <40).

Self-esteem was measured by the Culture-free Self-esteem Inventory (CFSEI) - Form B [27], a self-report scale that consists of 30 items measuring the individual’s perception about the self. The items are divided into two groups: items that indicate high self-esteem and those that reveal low self-esteem. The respondent checks either yes or no for each item. The form has four scales: General Self-Esteem, Academic Self-Esteem, Parental/Home Self-Esteem, and Social Self-Esteem. The items can be summed to produce a Global Self-Esteem Quotient score, in addition to the subscale scores, with higher scores indicating higher self-esteem.

Social adjustment was measured by a modified version of the Social Adjustment Scale–Self-Report (SAS-SR; [28]). This version is appropriate for school-age children and early adolescents and assesses social functioning over the past two months [29,30]. It consists of 36 items categorized into four scales of nine items each: (a) School, (b) Friends, (c) Family, and (d) Overall Adjustment. Each item is scored on a 5-point scale, with higher scores indicating worse functioning. A sum-score for each scale is produced representing the mean rating of all items categorized in the respective scale.

Family functioning was assessed by the Family Assessment Device (FAD) [31], which was administered to the caregivers of the patients. The family assessment device is a 60-item, self-administered instrument which is used to assess six dimensions of family functioning - Problem Solving, Communication, Roles, Affective Responsiveness, Affective Involvement, and Behavior Control. Additionally, a General Functioning scale assesses overall functioning of the family. Answers in the 60 items are as follows: “I strongly agree”, “I agree”, “I disagree”, “I strongly disagree”. Subscale scores range from 1 to 4. Lower scores suggest better functioning.

Patients’ emotional/behavioral problems were measured through the Youth Self-report (YSR) and Child Behavior Checklist (CBCL) questionnaires [32]. The YSR is a self-report questionnaire, administered to children and adolescents aged 11–18 years, while CBCL reflects parents’ perception of their children’s behavioral/emotional deficits. The CBCL is filled out by parents or surrogates of children aged 4 to 18 years. Both instruments contain 113 items rated on a three-point scale (i.e. 0 = not true, 1 = somewhat or sometimes true, 2 = very true or often true), based on the preceding six months. The following eight syndromes are scored, Anxious/Depressed, Withdrawn/ Depressed, Somatic Complaints, Social Problems, Thought Problems, Attention Problems, Delinquent Behavior, and Aggressive Behavior. Withdrawn/Depressed, Somatic Complaints, and Anxious/Depressed syndromes comprise an Internalizing group, related to disturbances of emotion or behavioral deficits and the Aggressive Behavior and Delinquent Behavior syndromes comprise an Externalizing group, considered to reflect conduct disorders or behavioral excess.

### Data analysis

Continuous variables are presented with mean ± standard deviation while quantitative variables are presented with absolute and relative frequencies. For the comparisons of proportions chi-square tests were used. Student’s t-tests were computed for the comparison of mean values. Differences in all study subscales according to age, sex, and the presence of CF were determined by the use of Multivariate Analysis of Variance (MANOVA). Logarithmic transformations were made for SAS-SR scales because they were not normally distributed. All *P* values reported are two-tailed. Statistical significance was set at 0.05 and analyses were conducted using SPSS statistical software (version 13.0).

## Results

The mean values for CFSEI, SAS-SR, and FAD scales for the control and CF group are described in Table 1. No significant differences were found for CFSEI scales between the two study groups. Furthermore, no gender or age differences were found for CFSEI scales defined by MANOVA in the CF and the control groups. Regarding SAS-SR dimensions, subjects with CF had significantly higher values (*P* < 0.05) for the Friends scale. Also significantly higher (*P* < 0.05) was the Overall Adjustment score of subjects with CF. Division of both patients and controls into two subgroups according to their age (8–11 and 12–18) revealed no differences in the SAS-SR scales. Also, division of both patients and controls in two subgroups according to the gender of the participant revealed no differences. Moreover, MANOVA indicated that there was no difference in the scores on FAD scales between the two groups. In the patient group (Table 2), parents of children aged 12–18 years (n = 19) scored significantly higher (*P* < 0.05) on all FAD scales except for Behavior Control compared to younger children aged 8–11 years (n = 17). No such differences were found in the control group. No gender differences were found in either group.

**Table 1:** Self-esteem, social adjustment, and family functioning of the patient and control groups

	**Controls**	**Cystic fibrosis**		
	**Mean ± SD**	**Mean ± SD**	***F *** **(df:1, 63 )**	***P *** MANOVA*
**CFSEI**				
General	7.7 ± 1.8	8.3 ± 1.4	1.21	0.276
Academic	4.1 ± 1.2	4.1 ± 1.1	0.00	0.949
Social	3.5 ± 0.8	3.2 ± 0.9	1.84	0.181
Parental/Home	4.5 ± 0.8	4.7 ± 0.6	0.38	0.540
Global quotient	20 ± 3	20.6 ± 2.8	0.56	0.458
**SAS-SR**				
Friends	2.1 ± 0.5	2.4 ± 0.5	6.74	0.012
School	1.7 ± 0.4	1.9 ± 0.6	3.78	0.056
Family	1.8 ± 0.4	1.8 ± 0.4	0.38	0.847
Overall adjustment	1.8 ± 0.3	2.0 ± 0.4	3.86	0.039
**FAD**				
Problem solving	1.7 ± 0.3	1.8 ± 0.5	0.43	0.514
Communication	1.8 ± 0.4	1.9 ± 0.5	0.23	0.631
Roles	2.2 ± 0.4	2.2 ± 0.4	0.04	0.841
Emotional responsiveness	1.8 ± 0.6	1.8 ± 0.6	0.00	0.953
Emotional involvement	1.9 ± 0.4	2.1 ± 0.4	2.24	0.140
Behavior control	2 ± 0.4	2.1 ± 0.5	0.77	0.385
General functioning	1.7 ± 0.4	1.7 ± 0.4	0.01	0.941

CFSEI, Culture-free Self-esteem Inventory; SAS-SR, Social Adjustment Scale–Self-Report; FAD**,** Family Assessment Device.

*sex and age were used as covariates.

**Table 2:** Family functioning of the patient group according to sex and age

	**Boys**	**Girls**			**8-11 years**	**12-18 years**		
**FAD**	**Mean ± SD**	**Mean ± SD**	**F (df:1,33)**	**P MANOVA**	**Mean ± SD**	**Mean ± SD**	**F (df:1,33)**	**P MANOVA**
Problem solving	1.7 ± 0.5	1.9 ± 0.6	0.01	0.939	1.5 ± 0.4	2 ± 0.6	4.88	0.035
Communication	1.8 ± 0.5	1.9 ± 0.5	0.13	0.724	1.6 ± 0.4	2.1 ± 0.4	8.79	0.006
Roles	2.2 ± 0.4	2.1 ± 0.4	1.51	0.230	2 ± 0.3	2.3 ± 0.4	5.41	0.027
Emotional responsiveness	1.7 ± 0.6	1.9 ± 0.6	0.07	0.790	1.5 ± 0.5	2 ± 0.6	4.98	0.034
Emotional involvement	2 ± 0.3	2.1 ± 0.5	0.04	0.840	1.9 ± 0.4	2.2 ± 0.3	4.51	0.043
Behavior control	2.1 ± 0.5	2.2 ± 0.4	0.09	0.769	1.9 ± 0.4	2.3 ± 0.4	3.64	0.067
General functioning	1.7 ± 0.4	1.9 ± 0.5	0.01	0.934	1.5 ± 0.4	2 ± 0.3	11.75	0.002

FAD, Family Assessment Device.

In Table 3 the differences in the mean values of YSR and CBCL scales of the patient and control groups are presented. No significant differences were found between groups for self-reports. On the contrary, parents of subjects with CF reported significantly higher scores on Withdrawn/Depressed, Thought Problems, and Delinquent Behavior scales (*P* ≤ 0.01) as compared to controls. No gender or age effects were found.

**Table 3:** Emotional/behavioral problems of the patient and control groups

	**YSR**				**CBCL**			
	**Controls**	**Cystic fibrosis**			**Controls**	**Cystic fibrosis**		
	**Mean ± SD**	**Mean ± SD**	***F *** **(df:1, 41 )**	***P *** MANOVA*	**Mean ± SD**	**Mean ± SD**	***F *** **(df:1, 63)**	***P *** MANOVA*
Withdrawn/Depressed	4 ± 2.3	4.3 ± 2.4	0.60	0.671	1.2 ± 1.2	2.5 ± 2.4	7.81	0.007
Anxious/Depressed	7.7 ± 4.1	6.8 ± 3.4	1.24	0.470	4 ± 3.6	5.2 ± 3.6	1.70	0.197
Somatic complaints	2.8 ± 2.2	3.4 ± 2.9	0.53	0.442	1 ± 1.3	1.6 ± 1.9	1.86	0.178
Social problems	2.6 ± 2.1	3.2 ± 1.5	2.85	0.272	2.3 ± 2.2	2.9 ± 2.1	1.21	0.276
Thought problems	2.8 ± 2.7	1.6 ± 1.8	0.01	0.100	0.2 ± 0.5	1.1 ± 1.7	6.98	0.011
Attention problems	5.9 ± 2.8	5.8 ± 2.8	0.05	0.928	3.6 ± 2.9	4.6 ± 3.6	1.48	0.228
Delinquent behavior	2.9 ± 2.4	2.7 ± 1.4	0.75	0.819	1.3 ± 1.5	2.5 ± 2	7.05	0.010
Aggressive behavior	9.6 ± 5.5	11.2 ± 6.1	0.01	0.393	5.3 ± 5.1	9.5 ± 6.9	7.50	0.008
Other problems	6.4 ± 3.6	6.3 ± 2.6	0.60	0.921	3.5 ± 3.2	6.2 ± 3.3	10.13	0.002

YSR, Youth Self-Report; CBCL, Child Behavior Checklist.

*sex and age were used as covariates.

## Discussion

The aim of the present study was the evaluation of family functioning, self-esteem, social adjustment, and the presence of psychopathology among Greek children and adolescents with CF compared to a group of healthy children and adolescents. No significant differences were found between the two groups in family functioning. There is evidence that families with children and adolescents with CF show resilience and do not differ from families with healthy members as far as their functioning level is concerned [33-35]. On the contrary, further validation of the CF group after its division in two distinct subgroups based on age (8–11 and 12–18) demonstrated statistically significant differences in almost all of the dimensions. More specifically, families with younger children demonstrate better functioning.

As children enter into adolescence, family relations become more difficult. Adolescence is a period of rapid social, cognitive, and physiological changes. Peers become more influential and family time reduces relative to time spent with peers [17]. Persistent movement towards increasing autonomy, need for separation from parents and adolescent-parents relationships, as well as attempts to challenge parental authority may lead to greater family conflict, less parent–child cohesion, and poorer communication [36,37]. One could argue that this finding could primarily be interpreted as a result of normal and expected tension in family relations secondary to adolescence.

On the other hand, chronic conditions amplify normal family issues. Adolescents with CF, despite their maturation and their normal tendency and need for autonomy, are kept dependent on their parents and members of their medical team [38,39]. In this context the patients have to face developmental challenges related to puberty, identity acquisition, and sexuality [15,16].

Additionally, age progression in CF is related to severity of illness and deterioration of health. CF typically worsens during adolescence and the presence of more frequent symptoms and pulmonary exacerbations correspond with greater illness burden [40]. Rising fears and fantasies may lead to emotional isolation and impede family communication.

No difference was found between the self-concept of healthy and CF children. Although there is evidence that chronic illness is a risk factor for low self-esteem [41], as far as patients with CF are concerned, there are previous relevant studies presenting results that are consistent with ours [42,43]. More specifically, research on the psychosocial characteristics of patients with chronic somatic conditions has revealed that adolescents with chronic illness perform lower levels of self-esteem than children with chronic illness [42], and so have similar studies focusing on adolescents with CF [44]. Issues related to small stature [18], delayed puberty [45], and deterioration of the symptoms [41] become more intense during adolescence for the young people with CF and may probably have a strong impact on self-esteem. In our study though, this fact could not be examined because of the restricted age range of the measure used (up to 13 years).

Children and adolescents with CF appear to have more difficulties in social adjustment when compared to their healthy peers. There is evidence that the presence of a childhood disability is associated with elevated use of health care services and the youth are four times as likely to be hospitalized and have eight times as many total hospital days as the general population [15]. More specifically, repeated absences and a lack of participation in recreational and sport activities, secondary to poor health, place children with CF at risk of social isolation due to diminished contacts and impaired leisure activities. Moreover, patients with CF must often be absent from school, resulting in delayed school performance and a sense of alienation [38].

Comparisons of the reports of children about their emotional/ behavioral problems showed no significant differences between the patients and controls in the present study. However, the parents of children with CF reported more emotional and behavioral difficulties in their offspring than did the parents of healthy children. Discrepancies between parents and children as informants have been reported before in studies of child psychopathology [6,26]. A possible interpretation for the discrepancy between the parents’ and children’s reports may relate to the fact that the parents of medically ill children are likely to be quite distressed, and as such may overestimate the externalizing behaviors (i.e., disobedience or other behavior problems) of their children or may underestimate their children’s ability to adapt to illness [46]. On the other hand, medically ill children may minimize and underreport their distress or symptoms as part of their adaptation to illness [47].

The present findings should be interpreted in the context of some limitations. First, the sample size was rather small, leading to a low statistical power. Second, patients with moderate or severe pulmonary impairment were under-represented in this sample. Therefore, this study did not manage to examine the relation of the medical background of the patients (e.g. pulmonary impairment, past history, or severity of respiratory infections) to dysfunction of social adjustment or other psychosocial outcomes. Third, the study did not examine the effect of socioeconomic status on the reported differences, threatening generalizability of the results. Lastly, the cross-sectional design of the study did not allow us to examine variations over time.

## Conclusions

The observed elevated levels of emotional/ behavioral problems and the difficulties in social adjustment of children and adolescents with CF indicate the need for the establishment of a collaborative treatment approach among medical and psychosocial personnel. Discrepancies between parents and children recommend the use of different sources of information. Future research should focus on the analysis of the variables that may mediate the effect on the psychosocial functioning of young patients and their families. Further investigation and analysis will be important to examine the relationship between advancing age, disease burden, and unfavorable psychosocial impact, as well as the different perceptions of children and their parents.

Families with children who suffer from CF make great effort to provide the best possible disease management, hoping to insure their children’s best quality and extension of life. A biopsychosocial approach should emphasize the assessment and treatment of the psychosocial distress of these patients alongside multiple somatic treatments.

## Competing interests

The authors declare that they have no competing interests.

## Authors’ contributions

KK conceptualized and carried out the study and drafted the manuscript. GG conceptualized and prepared the manuscript. SDi and SDo participated in the study design and patient screening and enrollment. SC and CB participated in the study design and drafted the manuscript. GK conceptualized and coordinated the study. All authors read and approved the final manuscript.
